# A population-based cross-sectional study of health service deficits among U.S. adults with depressive symptoms

**DOI:** 10.1186/1472-6963-13-160

**Published:** 2013-05-01

**Authors:** Krista L Huot, May Nawal Lutfiyya, Michael F Akers, Maria L Amaro, Michael T Swanoski, Sarah K Schweiss

**Affiliations:** 1 Essentia Health System, Ambulatory Care Pharmacy Services, Duluth, MN 55805, USA; 2 Essentia Institute of Rural Health, Duluth, MN 55803, USA; 3 University of Minnesota, College of Pharmacy, Ambulatory Care Residency Program, Minneapolis, MN 55455, USA

**Keywords:** Health service deficits, Depression, BRFSS, Pharmacy practice

## Abstract

**Background:**

Depression is a psychiatric condition that affects approximately one in five U.S. adults in their lifetime. No study that we know of has examined depressive symptoms and health service deficits in rural compared with non-rural populations. Four factors constitute the variable health service deficits: did not have health insurance, did not have a healthcare provider, deferred medical care because of cost and did not have a routine medical exam, all within the last 12 months. The aim of this study was to ascertain the prevalence of health service deficits in rural versus non-rural adults with depressive symptoms. Examining depressive symptoms by health service deficits is important because it allows us to approximate those with the condition who might not be receiving care for it. By analyzing national, population-based data, this study sought to fill in some important epidemiological gaps regarding depressive symptoms and health service deficits.

**Methods:**

For this analysis the population of interest was U.S. adults identified as currently having depressive symptoms using the PHQ-8 criteria. Behavior Risk Factor Surveillance Survey 2006 data were used in this analysis. Health service deficits was the primary dependent variable. Multivariate logistic regression analysis was performed to examine health service deficits experienced by adults with depression controlling for socioeconomic status, race and ethnicity and geographic locale (rural or non-rural).

**Results:**

Logistic regression analysis yielded that U.S. adults currently having depressive symptoms who were of low socioeconomic status, Hispanic ethnicity, or living in a rural locale were more likely to have at least one health service deficit.

**Conclusion:**

Analyzing data collected by a large surveillance system such as BRFSS, allows for an analysis incorporating an array of covariates not available from clinically-based data such as electronic health records. By identifying clinically depressed U.S. adults who also have at least one health service deficit, we were able to ascertain those most likely not receiving care for this debilitating condition. We believe community pharmacists are well suited to assist in connecting depressed, vulnerable populations with appropriate and needed care. This care would be best provided by an inter-professional team led by a primary care provider.

## Background

Depression is a psychiatric condition that affects approximately one in five U.S. adults in their lifetime and 121 million people worldwide [1-5]. In the U.S., this psychiatric disorder affects men and women disproportionately with a lifetime prevalence of 21.3% for women and 12.7% for men [6]. Some research has indicated that older adults have a higher prevalence of depression or depressive symptoms [4]. The trend for depression is increasing and it is projected that by 2020 depression will be among the top three contributors to the global disease burden [3,4,7].

Depression is a costly condition [8-10] with estimates of over $83 billion for total care costs, [8] and with over $10.4 billion for pharmaceutical costs. In 2009, Kessler, et al., reported that in the U.S. labor force, the annualized human capital loss to employers for major depressive disorder (MDD) was estimated to be greater than $36 billion [10].

There is no definitive diagnostic test for depression; it is diagnosed by presenting symptoms. The Patient Health Questionnaire depression scale (PHQ-9, PHQ-8) [11], a screening and diagnostic tool commonly used in primary care, incorporates the criteria of MDD based on the Diagnostic and Statistical Manual, Fourth Edition, Text Revision (DSM-IV-TR) criteria [12]. Based on the PHQ-9/8 score cut points, depression is divided into multiple levels: mild, moderate, moderately severe, and severe [11]. The symptoms of depression, as defined by the DSM-IV-TR, include: depressed mood, diminished interest or pleasure in activities, changes in appetite or weight, changes in sleep patterns, talking or moving slower or faster than usual, increased fatigue, loss of energy, feelings of worthlessness or guilt, inability to concentrate, and/or suicidal ideation. For a diagnosis of MDD, a person must have either of the first two symptoms listed above and a total of five or more symptoms present for at least 14 days.

Depression prevalence has been estimated for a number of different adult population groups in the U.S. such as race, [1,6,12,13] gender, [14] age, [4,7] and geographic locale (rural/non-rural residency) [14,15]. When examining geographic locale in relation to depression prevalence, many of the same lenses (race, gender, age) have been used. Nevertheless, few studies have examined the prevalence of depression between rural and non-rural adult populations. [13,16] Schoevers, et al. [15] found that all mood disorders, including depression, were significantly higher in non-rural areas; other researchers [13,16] aver that the prevalence of depression, specifically, is significantly higher in rural versus non-rural adult populations.

Additionally, no study that we know of has examined depressive symptoms and health service deficits in rural compared with non-rural populations. A *health service deficit* is an evolving analytic concept for use in health service-related research [17]. This concept facilitates the examination of how a group makes use of health services relevant to their condition. The four factors that constitute the variable health service deficits are: did not have health insurance, did not have a healthcare provider, deferred medical care because of cost and did not have a routine medical exam, all within the last 12 months [17].

In 2006 the Behavioral Risk Factor Surveillance Survey (BRFSS) included an optional module using PHQ-8 that was included by 33 states and territories in their annual surveillance survey. With the addition of this optional module in BRFSS, we were able to bring together our interest in depression, health service deficits, and geographic locale. The PHQ-8 screening and diagnostic tool was transformed into survey questions and all BRFSS respondents from the states that chose this optional module were asked these questions. This served to identify clinically depressed U.S. adults regardless of whether or not they had been previously diagnosed by a health care provider and regardless of whether or not they have ever received care for such. The aim of this study was to ascertain the prevalence of health service deficits in rural versus non-rural adults with symptoms of clinical depression. Examining symptoms of clinical depression by health service deficits is important because it allows us to approximate those with the condition who might not be receiving care for it. By identifying the demographic characteristics of those who might not be receiving care for symptoms of clinical depression a chance is presented to develop opportunities for care for those needing and not receiving it. By analyzing national, population-based data, this study sought to fill in some important epidemiological gaps regarding symptoms of clinical depression and health service deficits.

## Methods

For this study, 2006 Behavioral Risk Factor Surveillance Survey (BRFSS) data were analyzed to examine health service deficits as an important dimension of the epidemiology of depression and to ascertain if there were differences in the prevalence of health service deficits in rural versus non-rural adults with depression. The BRFSS survey is comprised of both core questions and optional modules. We chose this year of data to analyze because the optional BRFSS adult depression module was used by 33 states and/or territories. In the subsequent years of available data, many fewer states chose this option. We analyzed data collected by questions from both the core survey as well as the optional adult depression module based on the PHQ-8. Data analyses entailed both bivariate and multivariate techniques.

BRFFS data are collected using a random-digit dial telephone survey targeting adults 18 through 99 years of age. These data are collected under the guidance of the Centers for Disease Control and Prevention (CDC) in collaboration with all U.S. states and most U.S. territories. Once collected, BRFSS data are weighted by state or territory to represent the U.S. adult population. BRFSS data are cross-sectional and are focused on health risk factors and behaviors as well as chronic disease. A detailed description of the survey design and sampling measures can be found elsewhere [18].

For this analysis the population of interest was U.S. adults identified as currently experiencing symptoms of clinical depressed using the PHQ-8 criteria. All analyses were performed on weighted data as is recommended by the CDC. The weighting, calculated by the CDC uses the most recently available census data to provide a stratified representation of the nation’s non-institutionalized population.

In the analyses presented here a number of variables were either re-coded or computed. A summary of the re-coded variables used in the analyses for this study are presented in Table 1. The following three variables were computed: health service deficits, socioeconomic status and current depression.

**Table 1:** Original survey question and response categories with re-coded response categories 2006 BRFSS data

**Analysis variable**	**Survey question**	**Original response categories**		**Re-coded response categories**
**Sex**	Indicate sex of respondent.	Male		Male
		Female		Female
**Race and Ethnicity**	Which one of these groups would you say best represents your race?	Race responses were combined with Hispanic variable to create the second column categories		
		White	White, non-Hispanic	Caucasian
		Black or African American	Black non-Hispanic	African American
		Asian	Asian non-Hispanic	Other/multiracial
		Native Hawaiian or Other Pacific Islander	Native Hawaiian or Other Pacific Islander non-Hispanic	
		American Indian, Alaska Native	American Indian, Alaska Native non-Hispanic	
		Other	Other non-Hispanic	
		Multiracial but preferred race not asked	Multiracial non-Hispanic	
		Don’t know/Not sure, Refused	Don’t know/Not sure, Refused	Missing
	Are you Hispanic or Latino?	Yes	Hispanic	Hispanic
		No	Non-Hispanic	
		Don’t know/Not Sure, Refused	Don’t know/Not Sure, Refused	Missing
**Age Range**	What is your age?	_ _ age in years		18 – 29
				30 – 44
				45 - 64
				65 and older
**Education**	What is the highest grade or year of school you completed?	Never attended school or only kindergarten		<High School
		Grades 1 through 8 (Elementary)		
		Grades 9 through 11 (Some high school)		
		Grade 12 or GED (High school graduate)		Completed High School
		College 1 year to 3 years (Some college or technical school)		Educated Beyond High School
		College 4 years or more (College graduate)		
		Refused, Not asked or Missing		Missing
**Marital Status**	Are you: (marital status)	Married		Married or Living with Partner
		A member of an unmarried couple		
		Divorced		Unmarried and Not Living With a Partner
		Widowed		
		Separated		
		Never married		
		Refused, Not asked or Missing		Missing
**Household Income**	Is your annual household income from all sources:	Less than $10,000		Less than $25,000
		Less than $15,000 ($10,000 to less than $15,000)		
		Less than $20,000 ($15,000 to less than $20,000)		
		Less than $25,000 ($20,000 to less than $25,000)		
		Less than $35,000 ($25,000 to less than $35,000)		$25,000 to less than $50,000
		Less than $50,000 ($35,000 to less than $50,000)		
		Less than $75,000 ($50,000 to less than $75,000)		≥ $50,000
		$75,000 or more		
		Don’t know/Not sure, Refused and Not asked or Missing		Missing
**Have Health Insurance**	Do you have any kind of health care coverage, including health insurance, prepaid plans such as HMOs, or government plans such as Medicare?	Yes		Yes
		No		No
		Don’t know/Not Sure, Refused		Missing
**Have a Personal Physician**	Do you have one person you think of as your personal doctor or health care provider? (If “No” ask “Is there more than one or is there no person who you think of as your personal doctor or health care provider?”.)	Yes, only one		Yes
		More than one		
		No		No
		Don’t know/Not Sure, Refused, Not asked or Missing		Missing
**Timing of Last Routine Medical Check-up**	About how long has it been since you last visited a doctor for a routine checkup? [A routine checkup is a general physical exam, not an exam for a specific injury, illness, or condition.]	Within past year (anytime less than 12 months ago)		Within the Past 12 Months
		Within past 2 years (1 year but less than 2 years ago)		More than 12 Months Ago
		Within past 5 years (2 years but less than 5 years ago)		
		5 or more years ago		
		Never		
		Don’t know/Not sure or Refused		Missing
**Deferment of Medical Care Because of Cost**	Was there a time in the past 12 months when you needed to see a doctor but could not because of cost?	Yes		Yes
		No		No
		Don’t know/Not sure, Refused		Missing
**Self-Defined Health Status**	Would you say that in general your health is:	Excellent		Good to Excellent
		Very good		
		Good		
		Fair		Fair to Poor
		Poor		
		Don’t know/Not Sure, Refused, Not asked or Missing		Missing
**Residency by Geographic Locale**	Metropolitan Status Code	In the center city of an MSA		Non-rural
		Outside the center city of an MSA but inside the county containing the center city		
		Inside a suburban county of the MSA		
		In an MSA that has no center city		Rural
		Not in an MSA		
**Asthma Lifetime**	Have you ever been told by a doctor, nurse, or other health professional that you had asthma?	Yes		Yes
		No		No
		Don’t know/Not Sure		Missing
		Refused		
		Not asked or Missing		
**Diabetes**	Have you ever been told by a doctor that you have diabetes?	Yes		Have Diabetes
		Yes, but female told only during pregnancy		Do not Have Diabetes
		No		
		No, pre-diabetes or borderline diabetes		
		Don’t know/Not Sure		System Missing
		Refused		
		Not asked or Missing		
**CVD**	Has a doctor, nurse, or other health professional ever told you that you had any of the following? Angina or coronary heart disease.	Yes		Have CVD
		No		Do Not Have CVD
		Don’t know/Not Sure		System Missing
		Refused		
**Activity Limitation Due to Health Problems**	Are you limited in any way in any activities because of physical, mental, or emotional problems?	Yes		Have Limitations B/C Health
		No		Do not Have Health Related Limitations
		Don’t know/Not Sure		System Missing
		Refused		
**Children < =18 in Household**	How many children less than 18 years of age live in your household?	Number of childrenNotes: _ _ = Number of children		At Least One Child
		None		No Children
		Don’t know/Not Sure		System Missing
		Refused		
**Leisure Time Physical Activity**	Adults that report doing physical activity or exercise during the past 30 days other than their regular job	Had physical activity or exercise		Participated in leisure time PA
		No physical activity or exercise in last 30 days		Inactive
		Don’t know/Refused/Missing		System Missing
**Employment Status**	Are you currently:	Employed for wages		Employed
		Self-employed		
		Out of work for more than 1 year		Unemployed
		Out of work for less than 1 year		
		A homemaker		Not Working By Choice
		A student		
		Retired		
		Unable to work		Unable to Work
		Don’t know/Refused/Missing		System missing
**Get Needed Emotional Support**	How often do you get the social and emotional support you need?	Always		Sometimes to Always
		Usually		
		Sometimes		
		Rarely		Rarely to Never
		Never		
		Don’t know/Refused/Missing		System Missing
**Satisfaction with life**	In general, how satisfied are you with your life?	Very satisfied		Satisfied to Very Satisfied
		Satisfied		
		Dissatisfied		Dissatisfied to Very Dissatisfied
		Very dissatisfied		
		Don’t know/Refused/Missing		System Missing
**Smoking Status**	How often do you smoke?	smokes every day		smoker
		smokes some days		
		Former smoker		Non-Smoker
		Never smoked		
		Don’t know/Refused/Missing		System Missing
**BMI**	Calculated from height and weight	Neither overweight nor obese		Neither overweight nor obese
		Overweight		Overweight
		Obese		Obese
		Don’t know/Refused/Missing		System Missing
**Binge Drinking**	Binge drinkers (males having five or more drinks on one occasion, females having four or more drinks on one occasion)	No		Not a Binge Drinker
		Yes		Binge Drinker
		Don’t know/Refused/Missing		System Missing
**Heavy Alcohol Consumption**	Heavy drinkers (adult men having more than two drinks per day and adult women having more than one drink per day)	No		Not a Heavy Consumer of Alcohol
		Yes		Heavy Consumer of Alcohol
		Don’t know/Refused/Missing		System Missing
**Depression Lifetime**	Has a doctor or other healthcare provider EVER told you that you have a depressive disorder (including depression, major depression, dysthymia, or minor depression)?	Yes		Have depressive disorder
		No		Do not have depressive disorder
		Don’t know/Refused/Missing		System Missing

Health service deficits, the primary dependent variable in this analysis, was computed from the response categories of a number of different variables (health insurance status, personal healthcare provider, deferment of medical care because of cost, routine medical exam). These response categories were: did not have health insurance, did not have a healthcare provider, deferred medical care because of cost and did not have a routine medical exam, all within the last 12 months. Together these four issues form a constellation of conditions that can and often do lead to deficits in care in the U.S. health system. Without health insurance most people are unable to afford basic health care. Health insurance is also often the first line of access to having an identified primary care provider. Even with health insurance, the co-pay may be a hindrance, leading to the deferment of care because of cost. Also even with health insurance many do not have coverage for pharmacy costs. If and when a visit for healthcare entails the need for pharmacy, that additional cost may make the visit prohibitive. Finally, without a routine medical exam in the past 12 months, there is little chance that care for a chronic condition would be addressed. These four issues are somewhat interwoven and since health service deficits is an evolving concept they are given equal weight in this analysis. Having one of these constituted having a health service deficit.

Socioeconomic status (SES) was one of the primary independent variables. According to the World Health Organization (WHO) socioeconomic status is one of the strongest determinants of health [19]. While SES is a commonly used term in analyses across disciplines (e.g., sociology, social epidemiology, social psychology), many have noted that no general consensus exists about how to either define or measure the construct [20-22]. Typically SES refers to a combination of household income and other social measures such as attained educational level indexed into a single variable [20]. The most important purpose of SES is to provide some means of comparing relative position with regard to others. Almost always, SES is computed as a three-level variable (i.e., low, middle and high) [22]. Others have noted that various measures of SES are not interchangeable and that each one assesses a different aspect of SES and reflect the intent and approach of the investigator [22]. In our analyses, SES is a composite or computed variable comprised of two categorical variables: education and income. In keeping with convention, data categories from each of these individual variables were coded as one of low, mid-range or high and numbered 1, 2 or 3 respectively. The numbering was necessary in order to create a composite variable (in this instance one variable from two separate ones). The variables with numbered factors or categories were then added together to create the composite variable of SES. For education, low was less than high school and was coded as 1, mid-range was high school graduate and was coded as 2, and high was at least some college and was coded as 3. For income, low referred to the category < $25,000 and was coded as 1, mid-range referred to $25,000 - < $50,000 and was coded as 2, and high equaled ≥ $50,000 and was coded as 3. When the individual variables were added together the possible computed range was 2 – 6 points. These points were then indexed in the following manner: low = 2–3 points, mid-range = 4–5 points and high = 6 points. These cut-points were purposive. For the lowest range of the index, 2 points were the floor (smallest possible point assignment), for the mid-range of the index, 4 points was the floor and likewise for the high range of the index, 6 points was the floor. Any points below the floor for the mid-range were assigned to the lowest index category just as any points below the floor for the highest index category were assigned to the mid-range index category.

The standardized and validated PHQ-8 was used to measure current depression. This validated instrument consists of eight of the nine criteria on which the DSM-IV-TR diagnosis of depressive disorders is based [12]. The ninth question in the DSM-IV-TR assesses suicidal or self-injurious thoughts. It is omitted because interviewers/researchers are not able to provide adequate intervention by telephone if a respondent indicates that they are having such thoughts [23]. The PHQ-8 response set was standardized to make it similar to other BRFSS questions by asking the number of days in the past two weeks the respondent had experienced a particular depressive symptom. Similar to a methodology employed by other researchers (see Table 2), [17, 24] the modified response set was converted back to the original response set: 0 to 1 day = *not at all*, 2 to 6 days = *several days*, 7 to11 days = *more than half the days*, and 12 to 14 days = *nearly every day*, with points (0 to 3) assigned to each category, respectively. The scores for each item are summed to produce a total score between 0 and 24 points. A total score of 0 to 4 represents no significant depressive symptoms. A total score of 5 to 9 represents mild depressive symptoms; 10 to 14, moderate; 15 to 19, moderately severe; and 20 to 24, severe. This is summarized in Table 1. For our analyses, current depression was defined as: a PHQ-8 score of ≥ 10, which has an 88% sensitivity and an 88% specificity for major depression and, regardless of diagnostic status, typically represents clinically significant depression [17,23].

**Table 2:** **Patient health questionnaire (PHQ-8) scoring and Interpretation with BRFSS response conversion**[17,23]

**Over the *last 2 weeks, *how often have you been bothered by any of the following problems?**	**PHQ-8**	**Not at all**	**Several days**	**More than half the days**	**Nearly every day**
	**BFRSS conversion**	**0 - 1 day**	**2 - 6 days**	**7 - 11 days**	**12 - 14 day**
**1. **Little interest or pleasure in doing things		0	1	2	3
**2. **Feeling down, depressed, or hopeless		0	1	2	3
**3. **Trouble falling or staying asleep, or sleeping too much		0	1	2	3
**4. **Feeling tired or having little energy		0	1	2	3
**5. **Poor appetite or overeating		0	1	2	3
**6. **Feeling bad about yourself—or that you are a failure or have let yourself or your family down		0	1	2	3
**7. **Trouble concentrating on things, such as reading the newspaper or watching television		0	1	2	3
**8. **Moving or speaking so slowly that other people could have noticed. Or the opposite—being so fidgety or restless that you have been moving around a lot more than usual		0	1	2	3

Interpretation of Total Score/Total Score Depression Severity: 0–4 None, 5–9 Mild depression, 10–14 Moderate depression, 15–19 moderately severe depression, 20–24 severe depression.

The Metropolitan Statistical Area (MSA) variable included in BRFSS was used to define place of residence as either rural or non-rural. Rural residents were defined as persons living either within an MSA that had no city center or outside an MSA. Non-rural residents included all respondents living in a city center of an MSA, outside the city center of an MSA but inside the county containing the city center, or inside a suburban county of the MSA.

Race and ethnicity was calculated from participant responses to two separate survey questions—one regarding race and the other regarding Latino/Hispanic ethnicity. All race/ethnicity categories were computed as *mutually exclusive* entities. For example, all respondents coded as Caucasian chose white as their racial classification, likewise, black for African American, etc. If a respondent identified themselves as Hispanic or Latino they were classified by that ethnic category regardless of any additional racial classification. The category of other/Multiracial was also calculated and used in some of the analyses; however, it was not included in the bivariate analysis.

Bivariate analysis was performed to examine health service deficits for adults with depression by socioeconomic status, race/ethnicity, and geographic locales. Multivariate logistic regression analysis was performed to examine health service deficits experienced by adults with depression controlling for socioeconomic status, race and ethnicity and geographic locale.

We were interested in examining the distribution of health service deficit prevalence for depression by U.S. state. To identify the states where the greatest health service deficits for those with mental health issues including depression existed, we mapped the prevalence of health service deficits for U.S. adults with depression and other mental health issues by each state. This was done in this manner since depression prevalence data as calculated by PHQ-8 were not available for all states. The calculation using a different variable was required to formulate a proxy. For this proxy variable, we used responses to the following question in BRFSS: Now thinking about your mental health, which includes stress, depression, and problems with emotions, for how many days during the past 30 days was your mental health not good? We coded this variable into the bivariate categories of <14 days and > = 14 days. Fourteen days was chosen as the cut-point to coincide with the DSM-IV-TR diagnostic criteria for major depressive disorder. Furthermore, contingency table analysis (PHQ-8 by bad mental health days) was performed to examine the veracity of this proxy. Rating mental health as not good for > =14 days (in the last month) was predictive of current depression (OR = 9.408, 95% CI 9.391-9.426). ArcMap version 10.0 (ESRI, Redlands, CA) was used to map all U.S. states according to the health service deficits variable.

For all statistical analyses, alpha was set at p < 0.05. Statistical Package for Social Scientists (SPSS, IBM, Chicago, IL) version 19.0 was used to complete all statistical analyses performed for this study. Human subject approval was sought and received from Essentia Health’s Institutional Review Board (IRB).

## Results

The prevalence estimate for U.S. adults with current depression was 12.5%. While for lifetime depression it was 18.7%. Table 3, describing U.S Adults by depression status, displays the prevalence status of those currently depressed by each of the independent covariates used in the analysis. Notably, when stratifying by sociodemographic variables (sex, marital status, race/ethnicity, age, and employment status) the analysis yielded higher depression prevalence for women, unmarried/not partnered adults, African American and Other/Multiracial, middle aged adults (45–64 years), and those who are unable to work. When examining psychosocial covariates, depression prevalence was higher in individuals who rarely to never received needed emotional support, were dissatisfied to very dissatisfied with life, and/or were heavy drinkers.

**Table 3:** Description of U.S. adults > 18 years of age by depression status 2006 BRFSS (n = 65071506*)

**Variables**	**Factors**	% Not currently depressed (n = 59068733*)	% Currently depressed (n = 6002773*)
Sex	Male	93.0	7.0
	Female	89.4	10.6
Marital Status	Married or Living with a Partner	93.4	6.6
	Not Married or Partnered	87.1	12.9
Race and Ethnicity	Caucasian	91.5	8.5
	African American	87.9	12.1
	Hispanic	89.4	10.6
	Other/Multiracial	87.9	12.1
Age	18-29 Years	89.7	10.3
	30-44 Years	90.2	9.8
	45-64	89.2	10.8
	≥ 65 Years	94.6	5.4
Children < = 18 Years Living At Home	At Least One Child	90.3	9.7
	No Children	91.0	9.0
Employment Status	Employed	93.7	6.3
	Unemployed	76.9	23.1
	Not Working By Choice	93.5	6.5
	Unable To Work	56.4	43.6
Get Needed Emotional Support	Sometimes To Always	92.5	7.5
	Rarely To Never	71.4	28.6
Satisfied with Life	Satisfied To Very Satisfied	93.7	6.3
	Dissatisfied To Very Dissatisfied	41.3	58.7
Physical Activity	Exercised in Past 30 Days	93.5	6.5
	Inactive	82.3	17.7
Activity Limitations Because of Health	Do Not Have Limitations B/C Health	95.3	4.7
	Have Limitations B/C Health	76.6	23.4
Smoking Status	Current Smoker	81.4	18.6
	Non-Smoker	92.9	7.1
BMI	Neither Overweight Nor Obese	92.5	7.5
	Overweight	92.2	7.8
	Obese	86.1	13.9
Diabetes	Do Not Have Diabetes	91.4	8.6
	Have Diabetes	84.6	15.4
CVD	Do Not Have CVD	91.3	8.7
	Have CVD	82.6	17.4
Asthma	Do Not Have Asthma	92.1	7.9
	Have Asthma	82.1	17.9
Binge Drinking	Not A Binge Drinker	90.9	9.1
	Binge Drinker	90.1	9.9
Heavy Drinker	Not A Heavy Drinker	90.9	9.1
	Heavy Drinker	88.1	11.9
Geographic Locale	Non-Rural	91.1	8.9
	Rural	89.8	10.2
Health Service Deficits	No HSD	93.3	6.7
	At Least One HSD	87.5	12.5
Socioeconomic Status	Lower SES	81.2	18.8
	Middle SES	92.6	7.4
	High SES	96.9	3.1

* weighted n.

When viewing depression prevalence by a constellation of health status variables, those who were current smokers, had diabetes, CVD, and/or asthma had a higher prevalence of depression than those without these chronic conditions. Furthermore, those U.S adults who were obese, inactive, and reported having activity limitations because of health also had a higher prevalence of depression when compared to those without the aforementioned characteristics. When examining depression prevalence by the population covariates of geographic locale, health service deficits, and socioeconomic status, the prevalence of depression was higher in U.S. adults living in a rural locale, having at least one health service deficit, and being of low socioeconomic status.

Table 4 examines the relationship of socioeconomic status by geographic locale and race and ethnicity for depressed U.S adults with at least one health service deficit. The analysis indicated that, for all factors of geographic locale and all categories of race and ethnicity, there was a socioeconomic gradient for currently depressed adults with at least one health service deficit. This gradient follows the pattern of highest prevalence for all factors for both variables for lower socioeconomic status with lowest prevalence for high socioeconomic status.

**Table 4:** U.S. adults with current depression geographic locale and race and ethnicity by Socioeconomic Status 2006 BRFSS Data

**Variable**	**Factors**	**Socioeconomic status**		
		**% Lower SES**	**% Middle SES**	**% High SES**
**Geographic Locale**	Non-Rural	55.0	37.7	7.3
	Rural	65.6	31.1	3.3
**Race And Ethnicity**	Caucasian	53.0	39.7	7.3
	African American	72.5	24.4	3.2
	Hispanic	65.3	31.1	3.6
	Other/Multiracial	60.5	32.4	7.1

Table 5 displays the results of the logistic regression analysis performed. The population included in the analysis was U.S. adults with current depression. The dependent variable was *have at least one health service deficit.* Socioeconomic status, race and ethnicity, and geographic locale were the covariates entered into this model. When compared to high socioeconomic status, low socioeconomic status was the strongest predictor for U.S. adults with current depression having at least one health service deficit. In comparison to Caucasian adults with current depression, similar adults of Hispanic ethnicity had greater odds of having at least one health service deficit, while similar African Americans were less likely to have a health service deficit. Furthermore, those U.S. adults with current depression living in a rural locale were more likely to have at least one health service deficit than those living in a non-rural locale.

**Table 5:** Logistic regression U.S. adults with current depression with at least one health service deficit by race/ethnicity, socioeconomic status and geographic locale 2006 BRFSS data

**Independent variable and factors**		**Adjusted odds ratio (95.0% CI)**
Socioeconomic Status	Low SES	1.985 (1.972, 1.998)
	Middle SES	1.445 (1.435, 1.454)
	High SES	---*
Race And Ethnicity	African American	.954 (.949, .959)
	Hispanic	1.732 (1.722, 1.742)
	Other/Multiracial	1.267 (1.258, 1.276)
	Caucasian	---*
Geographic Locale	Rural	1.047 (1.043, 1.052)
	Non-Rural	---*

* reference category.

Table 6 displays the percent of all, low SES, Hispanic, and rural U.S. adults experiencing at least one health service deficit and self-reporting 14 or more days of mental health not good in past month by state. The ranges for each of these population groups varied from one another. For all U.S. adults, the percentage ranged from a low of 8.15% (Iowa) to a high of 20.78% (Kentucky). For low SES U.S. adults, the percentage ranged from a low of 12.24% (Iowa) to a high of 32.06% (Kentucky). For Hispanic U.S. adults, the percentage ranged from a low of 3.70% (Indiana) to a high of 29.45% (Pennsylvania). Lastly, for rural U.S. adults, the percentage ranged from a low of 8.01% (South Dakota) to a high of 24.32% (Kentucky). The widest percentage range was for U.S. Hispanic adults, followed in order by low SES and rural adults.

**Table 6:** Percent of all, low SES, Hispanic, and rural U.S. adults experiencing at least one health service deficit* and reporting 14 or more days mental health not good in past month by state of residence 2006 BRFSS data

**States**	**% All US adults**	**% Low SES**	**% Hispanic**	**% Rural**
Alabama	19.01	26.60	23.14	19.28
Alaska	10.46	17.75	13.86	9.24
Arizona	12.00	16.84	10.40	14.83
Arkansas	16.64	25.92	13.41	17.39
California	13.70	20.21	15.54	14.19
Colorado	11.16	19.50	12.49	11.22
Connecticut	10.97	18.47	15.92	9.83
Delaware	13.21	22.17	16.26	13.83
DC	9.49	18.30	7.75	n/a
Florida	14.45	19.15	12.26	13.23
Georgia	13.50	21.85	12.44	14.85
Hawaii	9.83	18.73	13.92	10.80
Idaho	13.40	19.78	10.78	12.56
Illinois	11.44	18.86	10.91	10.86
Indiana	14.18	22.53	3.70	13.30
Iowa	8.15	12.24	7.93	8.13
Kansas	11.62	19.90	11.06	11.53
Kentucky	20.78	32.06	16.46	24.32
Louisiana	13.52	18.10	7.57	12.77
Maine	13.64	20.46	21.68	12.81
Maryland	12.10	25.68	19.88	16.59
Massachusetts	13.09	26.69	14.31	12.05
Michigan	14.93	23.14	23.81	13.61
Minnesota	9.00	16.73	9.66	9.21
Mississippi	19.17	26.41	23.92	19.46
Missouri	15.12	25.21	22.71	16.25
Montana	10.89	20.42	11.75	10.01
Nebraska	9.96	14.44	7.42	9.82
Nevada	13.29	18.90	10.42	15.26
New Hampshire	13.13	24.05	8.59	12.85
New Jersey	12.51	21.31	11.90	n/a
New Mexico	12.01	15.57	12.88	13.24
New York	10.76	16.76	10.39	11.31
North Carolina	14.30	20.29	7.92	14.99
North Dakota	8.32	14.62	8.81	8.18
Ohio	14.71	26.48	13.53	12.87
Oklahoma	16.48	24.42	15.52	15.57
Oregon	10.43	16.94	8.46	9.83
Pennsylvania	13.71	21.92	29.45	13.81
Rhode Island	14.31	23.47	12.20	n/a
South Carolina	14.51	21.94	11.45	15.37
South Dakota	8.54	16.09	11.10	8.01
Tennessee	14.15	23.07	10.81	13.18
Texas	12.98	19.80	9.50	14.36
Utah	10.55	19.41	10.66	9.22
Vermont	11.15	18.77	20.11	11.34
Virginia	12.23	23.96	18.05	17.17
Washington	12.16	21.23	11.33	11.98
West Virginia	18.81	27.23	21.28	19.65
Wisconsin	11.41	23.33	15.72	12.66
Wyoming	11.01	17.31	13.26	10.96
US Total	13.31	21.17	12.68	13.86

*In past 12 months at least one of: no health insurance, no HCP, deferred medical care because of cost, no routine medical exam.

**Figure 1:**
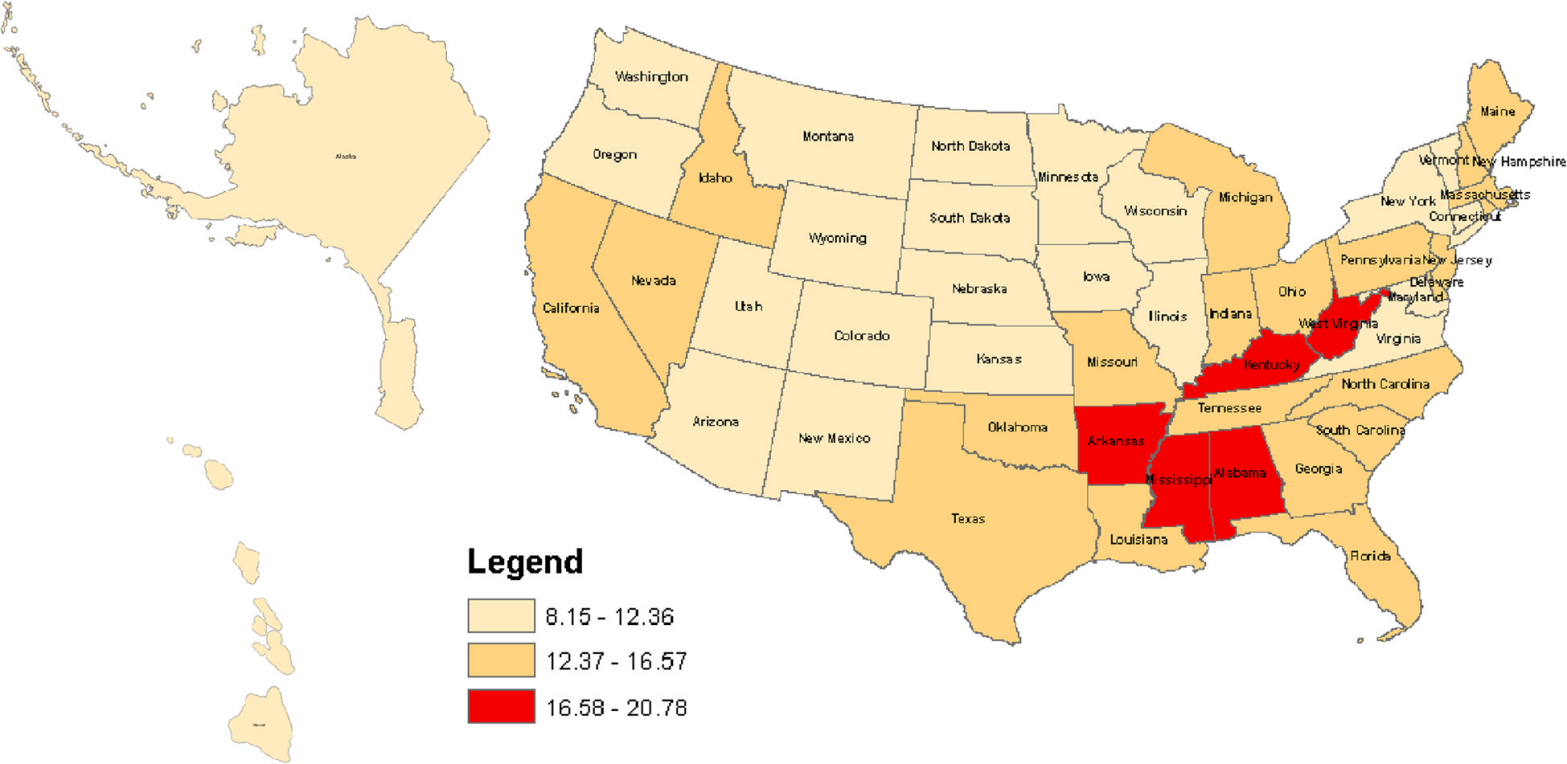
Percent of all U.S. adults with at least one health service deficit* self-reporting 14 or greater bad mental health days. *one of: no health insurance, no health care provider, no routine medical check-up, deferment of medical care because of cost.

**Figure 2:**
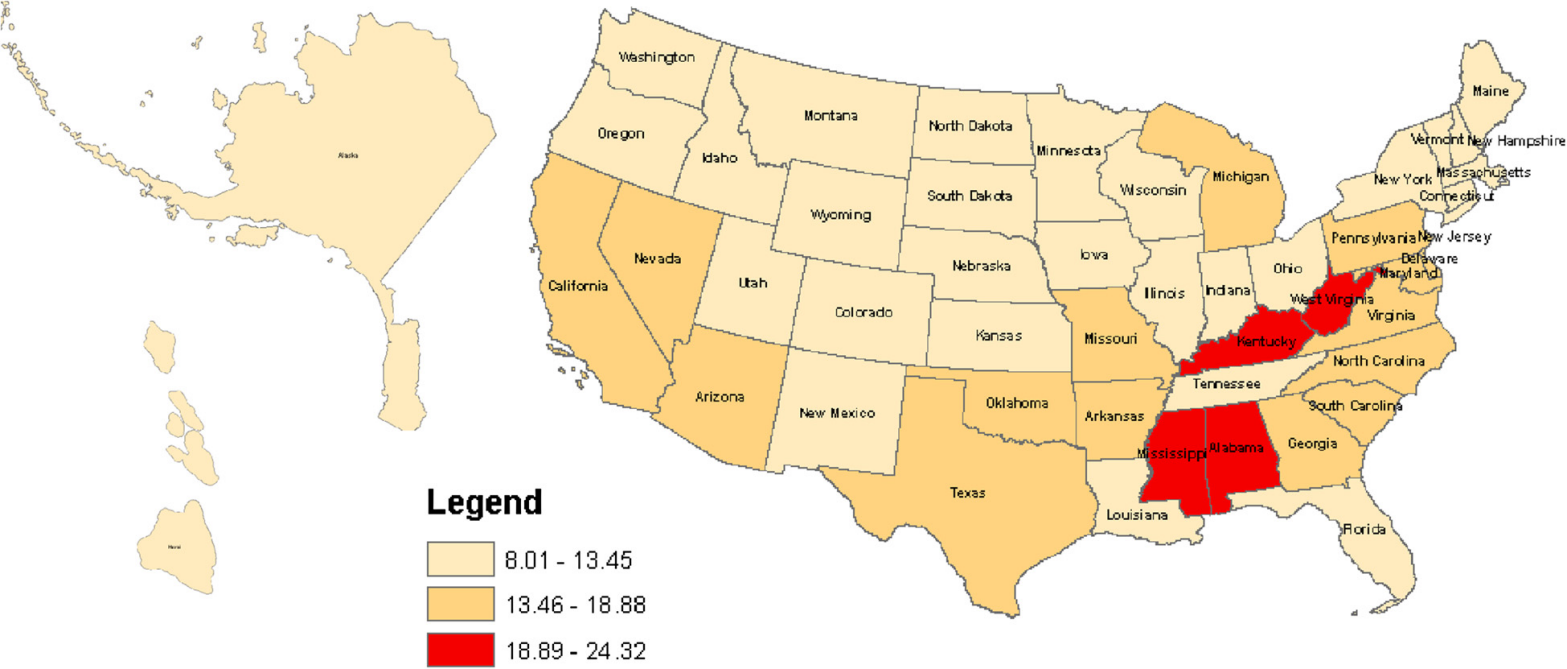
Percent of rural U.S. adults with at least one health service deficit* self-reporting 14 or greater bad mental health days. *one of: no health insurance, no health care provider, no routine medical check-up, deferment of medical care because of cost.

**Figure 3:**
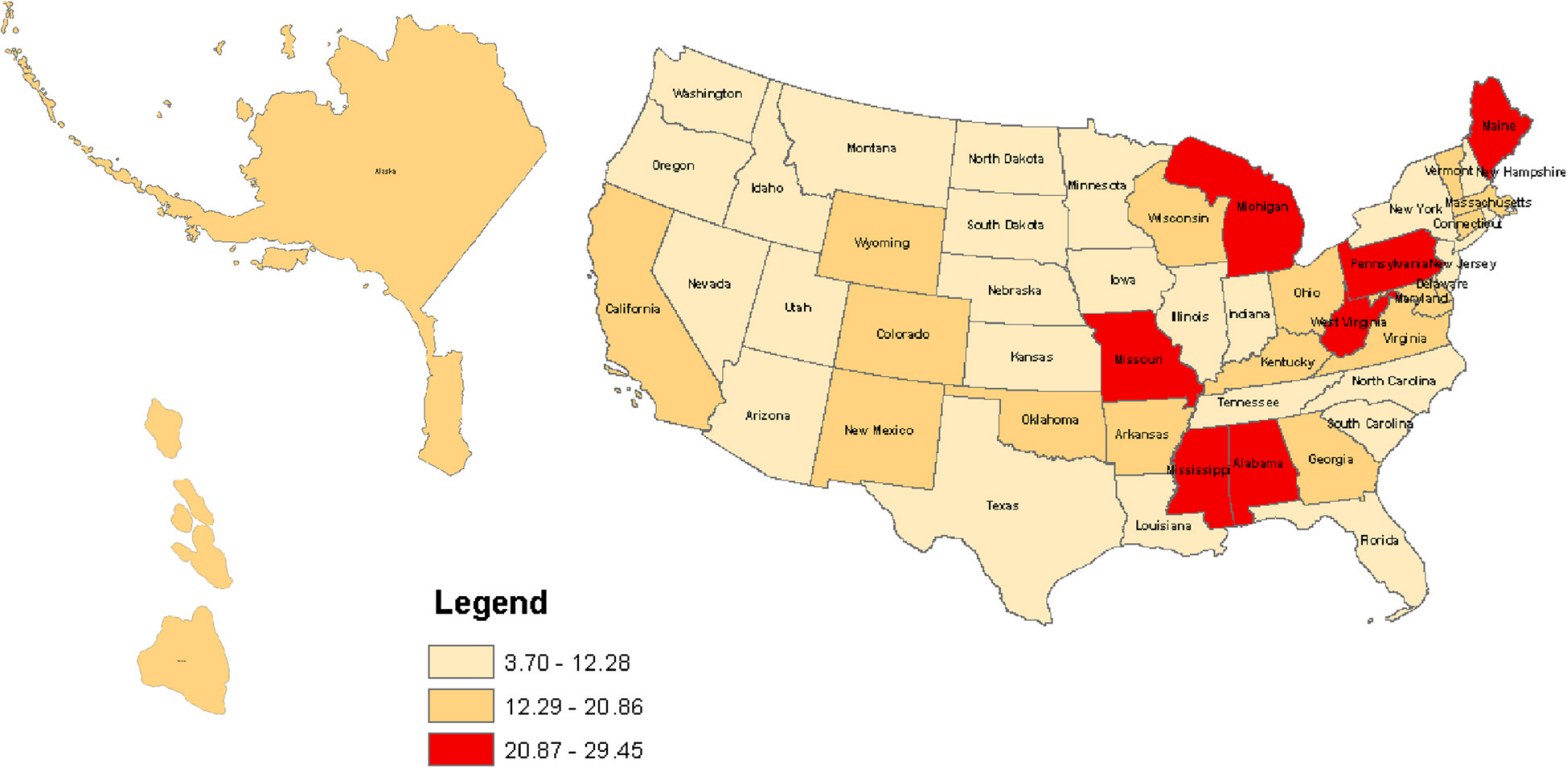
Percent of Hispanic U.S. adults with at least one health service deficit* self-reporting 14 or greater bad mental health days.

**Figure 4:**
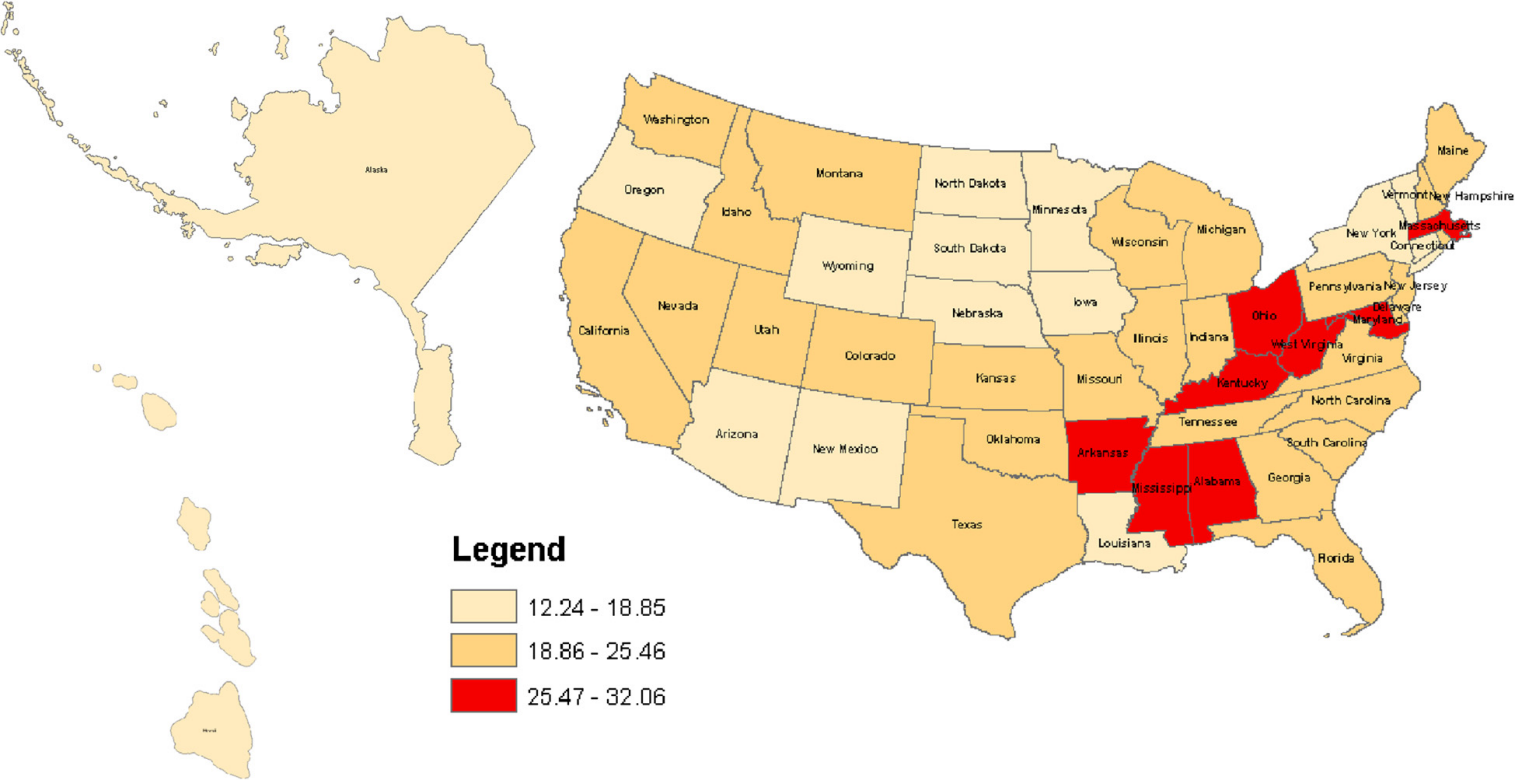
Percent of low SES U.S. adults with at least one health service deficit* self-reporting 14 or greater bad mental health days. *one of: no health insurance, no health care provider, no routine medical check-up, deferment of medical care because of cost.

Figures 1, 2, 3 and 4 geographically display by state the percent ranges of U.S. adults with at least one health service deficit reporting 14 or greater bad mental health days by tertiles. For all U.S. adults the highest tertile ranged from 16.58% to 20.78% and included the following states: Alabama, Arkansas, Kentucky, Mississippi, and West Virginia. For rural U.S adults the highest tertile ranged from 18.89% to 24.32% and included: Alabama, Kentucky, Mississippi, and West Virginia. For Hispanic U.S. adults the highest tertile ranged from 20.87% to 29.45% and included the following states: Alabama, Maine, Michigan, Mississippi, Missouri, Pennsylvania, and West Virginia. Lastly, for low SES adults, the highest tertile ranged from 25.47% to 32.06% and included: Alabama, Arkansas, Kentucky, Maryland, Massachusetts, Mississippi, Ohio, and West Virginia. For low SES U.S. adults, the floor for the highest tertile range (25.47% to 32.06%) was higher than the ceiling of the highest tertile range for all and rural U.S. adults, but not for Hispanic U.S. adults.

## Discussion

Using BRFSS data collected in a non-clinical setting, the estimates for current and lifetime depression derived from this study reflected those previously reported in the literature [1-6]. Analyzing data collected by a large surveillance system such as BRFSS, allows for an analysis incorporating a large array of covariates not available when analyzing clinically-based data. For instance, our analysis also revealed that by employment status, 23.1% of those U.S. adults who were unemployed were currently depressed, and 43.6% of those who were unable to work were also currently depressed. Additionally, 58.7% of U.S. adults self-reporting that they were dissatisfied to very dissatisfied with life were currently depressed. Moreover, of those of lower SES, 18.8% were currently depressed in comparison to 3.1% of high SES U.S. adults. These findings speak to the advantage of using large surveillance system data. Medical record data alone typically does not include a broad scope of covariates that can be analyzed to flesh out a detailed epidemiological picture. By examining depression by health service deficits we are, in part, identifying a population suffering from this condition that is not receiving care for it and hence would not be included in health record data.

The socioeconomic gradient yielded for depression prevalence for those with a health service deficit would also not be identifiable from most health records. Again, those with health service deficits are most likely not receiving care for depression and as our analysis has revealed these are most likely more vulnerable groups of U.S. adults (e.g. low SES, Hispanic ethnicity, and rural residents). Hispanic ethnicity emerged as an independent risk factor for health service deficits for U.S. adults with depressive symptoms as did other/multiracial. Rural residency emerged (albeit modestly) as an independent risk factor for health service deficits for U.S. adults with depressive symptoms.

Identifying those with depression and health service deficits helps locate the need for targeted interventions. Because these interventions would be targeting U.S. adults with a health service deficit, they are likely not going to be associated with a hospital or clinic. For this reason, community pharmacists are well positioned to initiate interventions that are inter-professional in nature involving primary care, behavioral health, and pharmacy. A primary care practitioner would need to be involved in the assessment, diagnosis, and prescribing pharmacotherapy. A behavioral health professional would need to be involved for counseling. Pharmacists would be a key player in supporting the intervention by connecting adults with health service deficits and depressive symptoms with patient assistance programs to provide access to affordable medications. Furthermore, pharmacists could build a professional relationship with these patients in order to provide assure effectiveness of therapy and monitor for adverse effects.

The intervention needs to target not only this population, but the family and friends of those in need of care. To accomplish this, community pharmacists could focus their efforts in “high traffic” areas such as grocery stores or community pharmacies, particularly those located in rural, Hispanic, and low socioeconomic communities. This intervention would include public advertisement of the signs and symptoms of depression, followed by offering an event where practitioners would be available for consultation, assessment, and initial treatment. Care would need to be taken to protect the privacy of any and all potential patients attending the therapeutic event. As a result of the event, the goal would be to help new patients gain access to services available for vulnerable populations.

While states were not our unit of analysis for this study we did assess the distribution of health service deficits for U.S. adults with mental health issues (14 or more bad mental health days in a 30 day period) by U.S. state and Hispanic ethnicity, low SES and rural residency. The disparities across and between states are interesting and warrant further investigation. Presently they are beyond the scope of this paper.

### Study limitations

Several potential limitations to this study should be noted. First, the survey is based on telephone derived data and may lack representation because those who could not be reached by phone could not participate in the survey. For instance, persons of lower socioeconomic status may have been excluded because of poorer phone access. Widespread use of answering machines and caller identification now allow people to filter their phone calls potentially leading to a passive refusal to participate in surveys such as the BRFSS. Nevertheless, call filtering is beyond the control of survey administrators and the vast majority of U.S. residents live in households with telephones, which minimizes the bias of lack of phone access. Additionally, U.S. cell phone numbers are now included in the pool of phones contacted for the survey ensuring the widest possible net being cast. Study strength is in the use of a national database that included a robust sample of residents weighted to reflect the demographics of the U.S. population.

A second limitation is that the survey used close-ended questions, which limit participants’ options to fully explain response choices. Nonetheless, the survey questions were worded such that the answer choices covered a wide range of response possibilities. A third, and related, limitation is that the answers are self-reported, which introduces the possibility of recall bias on the part of the survey participants. Furthermore, this study did not account for seasonal depression or seasonal depressive symptoms, which may have been manifested at the time of the call because of the time of year and been mistaken for major depressive disorder. This limitation is beyond the control of the study. A final potential bias resulted from the languages of the survey – English and Spanish. Individuals who did not speak English or Spanish were excluded from this survey.

## Conclusion

By identifying clinically depressed U.S. adults who also have at least one health service deficit, we were able to ascertain populations most likely not receiving care for this debilitating condition. We believe community pharmacists are well suited to assist in connecting depressed, vulnerable populations with appropriate and needed care. This care would be best provided by an inter-professional team led by a primary care provider.

## Competing interests

The authors declare that they have no competing interests.

## Authors’ contribution

All authors contributed equally to the conception of this paper. MNL carried out all of the statistical analyses. KLH, MLA, MKA, SS interpreted the analyzed data. KLH, MNL, MLA, MKA, MTS wrote the first draft of the manuscript. All authors refined the manuscript in multiple drafts before submission. All authors read and approved the final manuscript.

## Pre-publication history

The pre-publication history for this paper can be accessed here:

http://www.biomedcentral.com/1472-6963/13/160/prepub
